# Prevalence of extramammary Paget’s disease in urban China: a population-based study

**DOI:** 10.1186/s13023-021-01715-6

**Published:** 2021-03-17

**Authors:** Shilu Yin, Lu Xu, Shengfeng Wang, Jingnan Feng, Lili Liu, Guozhen Liu, Jinxi Wang, Siyan Zhan, Zhenmin Zhao, Pei Gao

**Affiliations:** 1 Department of Plastic Surgery, Peking University Third Hospital, 49 North Garden Road, Haidian District, Beijing, 100191 China; 2 Department of Epidemiology and Biostatistics, School of Public Health, Peking University, 38 Xueyuan Road, Haidian District, Beijing, 100191 China; 3 Peking University Health Information Technology Co. Ltd, 52 North Fourth Ring West Road, Haidian District, Beijing, 100080 China; 4 Shanghai Songsheng Business Consulting Co. Ltd, 6 Chaoyang Men North street, Dongcheng District, Beijing, 100000 China; 5 Research Center of Clinical Epidemiology, Peking University Third Hospital, 49 North Garden Road, Haidian District, Beijing, 100191 China; 6 Center for Intelligent Public Health, Institute for Artificial Intelligence, Peking University, 38 Xueyuan Road, Haidian District, Beijing, 100191 China

**Keywords:** Extramammary Paget’s disease, Prevalence, Epidemiology, Claim data, Population-based study

## Abstract

**Background:**

Extramammary Paget’s disease (EMPD) is an intraepithelial adenocarcinoma. The chronic relapsing clinical course and unbearable clinical symptoms of extramammary Paget’s disease usually result in a markedly diminished quality of life. No national data are available on descriptive epidemiology of EMPD in China, the most populous country over the world. This population-based study aimed to estimate the prevalence and associated sex and age patterns of EMPD in China.

**Methods:**

This study was conducted using data from China’s Urban Employee Basic Medical Insurance and Urban Resident Basic Medical Insurance, covering approximately 0.43 billion Chinese urban residents in 2016. Patients with EMPD were identified based on the diagnostic names and codes in claim data.

**Results:**

A total of 53 males and 31 females with EMPD were found. The crude prevalence in 2016 was 0.04 per 100,000 population [95% confidence interval (CI) 0.02–0.06], ranging from 0.01 (95% CI 0.00–0.02) in North or Northeast China to 0.08 (95% CI 0.03–0.16) in Southwest China. The rate was higher in males (0.05, 95% CI 0.03–0.08) compared with females (0.03, 95% CI 0.02–0.05). The mean age of patients was 65.87 (standard deviation: 14.21) years, with the peak prevalence appeared in patients aged 70–79 (0.28, 95% CI 0.16–0.42).

**Conclusions:**

The prevalence of EMPD was markedly lower than those in the United States and Europe, and varied across regions in China. Chinese patients were much younger, with significant male predominance. Further studies are warranted to examine potential pathophysiologic mechanism.

## Background

Extramammary Paget’s disease (EMPD) is an intraepithelial adenocarcinoma which predominantly affects areas with a high apocrine gland concentration, including the vulva, perineal, perianal, scrotal, penile and axillae skin [1–3]. EMPD lesion is notorious for its chronic relapsing clinical course and unbearable clinical symptoms such as itching, skin ulcer and bleeding [2, 4]. The standard treatment for EMPD is often extensive surgical excision followed by plastic reconstruction, which frequently leaves anatomical and functional impairments, and results in a significantly diminished quality of life [4, 5]. Understanding the epidemiological characteristics of EMPD is critical to inform policy making about EMPD management. However, epidemiological information of EMPD was limited, especially in the developing countries including China.

EMPD was reported to be most common in individuals between 60 and 80 years of age, with a peak incidence at 65 years [2, 6–8]. In western literatures, a female predominance was reported in aged Caucasians [9–13]. By contrast, a male predominance was found by both single- and multicenter studies in China, Japan, and Korea, with a male to female ratio between 2:1 and 5:1 [14–17]. EMPD is rare, with the reported mean incidence rate no more than 0.11 patients per 100,000 person-years in Europe [12, 13, 18]. In multiracial country like the United States, the incidence of EMPD showed ethnic disparity, the incidence in black males was nearly four times lower and in Asians/Pacific islander males four times higher than that in Caucasian males between 1973 and 2009 [1]. No national epidemiological studies are available to estimate the prevalence and incidence of EMPD in Asian countries, including China.

This study was conducted to provide recent estimates of the prevalence of EMPD in mainland China and to investigate its patterns across sexes, age groups and geographical regions, using a nationally representative data in 2016.

## Materials and methods

### Data sources

The anonymous claim data we used were from Urban Employee Basic Medical Insurance (UEBMI) and Urban Residence Basic Medical Insurance (URBMI). As two main health insurance schemes in urban China, UEBMI is oriented to working or retired employees and URBMI is for urban residents unemployed. By 2016, UEBMI and URBMI have covered approximately 95% of the urban population in China. Every city updated the data in both databases on a monthly basis. The reimbursement records of the insured population will be recorded in the database, no matter the proportion they paid for the medical service. Alongside the fees and procedures claimed, diagnostic information (i.e., disease names and disease codes) and sociodemographic characteristics (i.e., ethnicity, sex, birth date, place of residence, etc.) are recorded as well.

### Study population

This national population-based study used the UEBMI and URBMI data of 23 provinces between January 1st, 2016 and December 31st, 2016. Eight provinces including Beijing, Shanghai, Sichuan, Ningxia, Hebei, Tianjin, Fujian and Tibet were excluded due to absence or abnormality of key information such as diagnostic information, only containing a single insurance type or reporting policy exemptions. The study protocol was approved by the ethical review committee of the Peking University Health Science Center (IRB. No.: IRB00001052-18012), and the informed consent requirement was waived.

### Case identification

The identification of patients with EMPD was based on the diagnostic information in the database, such as diagnostic text and International Classification of Diseases (ICD) code. Natural language processing was utilized to normalize the diagnostic information with a dictionary of potential EMPD defined by prestigious clinicians. Potential patients with EMPD were selected by ICD-10 (C51.902, C50.903, M85400/3, C63.252, M85420/3, M85420/6) and Chinese medical terms of diseases including EMPD, Paget's disease of perineum, Paget's disease of pubic caruncle, Paget's disease of scrotum, Paget's disease of penis, extramammary eczematoid carcinoma, extramammary eczematoid carcinoma of perineum, extramammary eczematoid carcinoma of pubic caruncle, extramammary eczematoid carcinoma of scrotum and extramammary eczematoid carcinoma of penis. Two prestigious clinicians read the diagnostic information of each potential patient with EMPD independently to identify actual target patients. Any disagreements between them would be judged by another senior clinician.

### Statistical analysis

Through a two-stage approach [19], the national prevalence of EMPD in 2016 was calculated. In the first stage, the prevalence in each province was estimated. The denominator (*N*) was the number of insured people from each province in the database in 2016. The numerator (*M)* was the estimated number of patients with EMPD from each province, considering the existence of claim records with missing diagnostic information. The insured people from each province can be divided into three groups: those without claim records (*N*_*1*_), those with complete diagnostic information in their claim records (*N*_*2*_), and those with claim records but without diagnostic information (*N*_*3*_). The patients with EMPD (*M*_*2*_) that we actually observed were from *N*_*2*_. However, a proportion of patients with EMPD (*M*_*3*_) were in *N*_*3*_. Since the missing diagnostic information in the database was mainly due to the administrative issues at prefecture-level cities, we assumed that the probability of having EMPD was not associated with the missing status of diagnostic information i.e. *M* was considered as (*N*_*2*_ + *N*_*3*_) *M*_*2*_/*N*_*2*_. In the second stage, we pooled the prevalence of each province using a random effects meta-analysis to calculate the national or regional prevalence, in which the Freeman–Tukey double arcsine transformation was used to stabilize the variance of province-level prevalence.

The prevalence of EMPD was estimated by sex, age, and geographical region (East, North, Northeast, Northwest, Southcentral, and Southwest) as well. To test the robustness of the main results, sensitivity analyses were conducted by setting a stricter algorithm (not considering the patients with diagnostic information containing eczematoid carcinoma) to identify actual patients with EMPD, by only considering the observed patients with EMPD to estimate the lower bound of the prevalence, and by excluding the top 10% of provinces with missing diagnostic information. Based on the 2010 Chinese national census data, the Revised European Standard Population (RESP) 2013, the 2010 US population and the 2011 Australian population, age-adjusted rates of EMPD prevalence were provided. Prevalence with 95% confidence interval (CI) was estimated by Poisson distribution. Student's *t* test was used for continuous variables and the chi-squared test was used for categorical variables. All statistical analyses were done by Stata version 15.0, and a two-sided *P* value < 0.05 was used to indicate statistically significant differences in basic characteristics between male and female patients with EMPD.

## Results

Approximately 0.43 billion individuals were included in this study (Table 1). There were 84 patients with EMPD in the database in total (Table 1). The mean age of the observed patients with EMPD was 65.87 [standard deviation (SD): 14.21] years. There were no statistically significant differences in mean age and area distribution between male and female patients with EMPD (Tables 1, 2).

**Table 1:** Age distribution of the study population and patients with EMPD from urban China in 2016

Characteristic		Study population (million)			Patients with EMPD			
		Total	Male	Female	Total	Male	Female	*P* value
Total number		425.83	221.86	203.97	84	53	31	
Age (years)	Mean (SD)	37.68 (20.00)	37.21 (14.31)	38.20 (13.96)	65.87 (14.21)	67.98 (12.64)	62.26 (16.13)	.08
Age groups, No. (%)	0–17	68.3 (16.04)	36.8 (16.59)	31.5 (15.44)	0 (0)	0 (0)	0 (0)	.04
	18–39	167.2 (39.26)	87.7 (39.53)	79.48 (38.97)	4 (4.76)	1 (1.89)	3 (9.68)	
	40–59	123.1 (28.91)	63.79 (28.75)	59.31 (29.08)	18 (21.43)	12 (22.64)	6 (19.35)	
	60–69	37.89 (8.9)	19.42 (8.75)	18.47 (9.05)	26 (30.95)	12 (22.64)	14 (45.16)	
	70–79	19 (4.46)	9.51 (4.29)	9.48 (4.65)	23 (27.38)	19 (35.85)	4 (12.90)	
	≥ 80	10.35 (2.43)	4.63 (2.09)	5.72 (2.8)	13 (15.48)	9 (16.98)	4 (12.90)	

*EMPD* extramammary Paget's disease, *SD* standard deviation

**Table 2:** Area distribution of the study population and patients with EMPD from urban China in 2016

Characteristic	Study population (million)			Patients with EMPD			
	Total	Male	Female	Total	Male	Female	*P* value
Area^a^, No. (%)							
East	171.30 (40.24)	88.68 (39.97)	82.67 (40.53)	29 (34.52)	17 (32.08)	12 (38.71)	.38
North	18.92 (4.44)	9.63 (4.34)	9.28 (4.55)	0 (0)	0 (0)	0 (0)	
Northeast	42.87 (10.07)	21.51 (9.69)	21.36 (10.47)	1 (1.19)	0 (0)	1 (3.23)	
Northwest	21.60 (5.07)	11.25 (5.07)	10.35 (5.07)	8 (9.52)	6 (11.32)	2 (6.45)	
Southcentral	123.60 (29.02)	66.18 (29.83)	57.41 (28.15)	31 (36.90)	22 (41.51)	9 (29.03)	
Southwest	47.50 (11.15)	24.61 (11.09)	22.89 (11.22)	15 (17.86)	8 (15.09)	7 (22.58)	

*EMPD* extramammary Paget's disease

^a^East area contains Jiangsu, Zhejiang, Anhui, Jiangxi, and Shandong (five provinces). North area contains Shanxi, Inner Mongolia (two provinces). Northeast contains Liaoning, Jilin, Heilongjiang (three provinces). Northwest contains Shaanxi, Qinghai, Gansu, and Xinjiang (four provinces). Southcentral contains Henan, Hubei, Hunan, Guangdong, Guangxi and Hainan (six provinces). Southwest contains Chongqing, Guizhou and Yunnan (three provinces)

### Prevalence

**Fig. 1:**
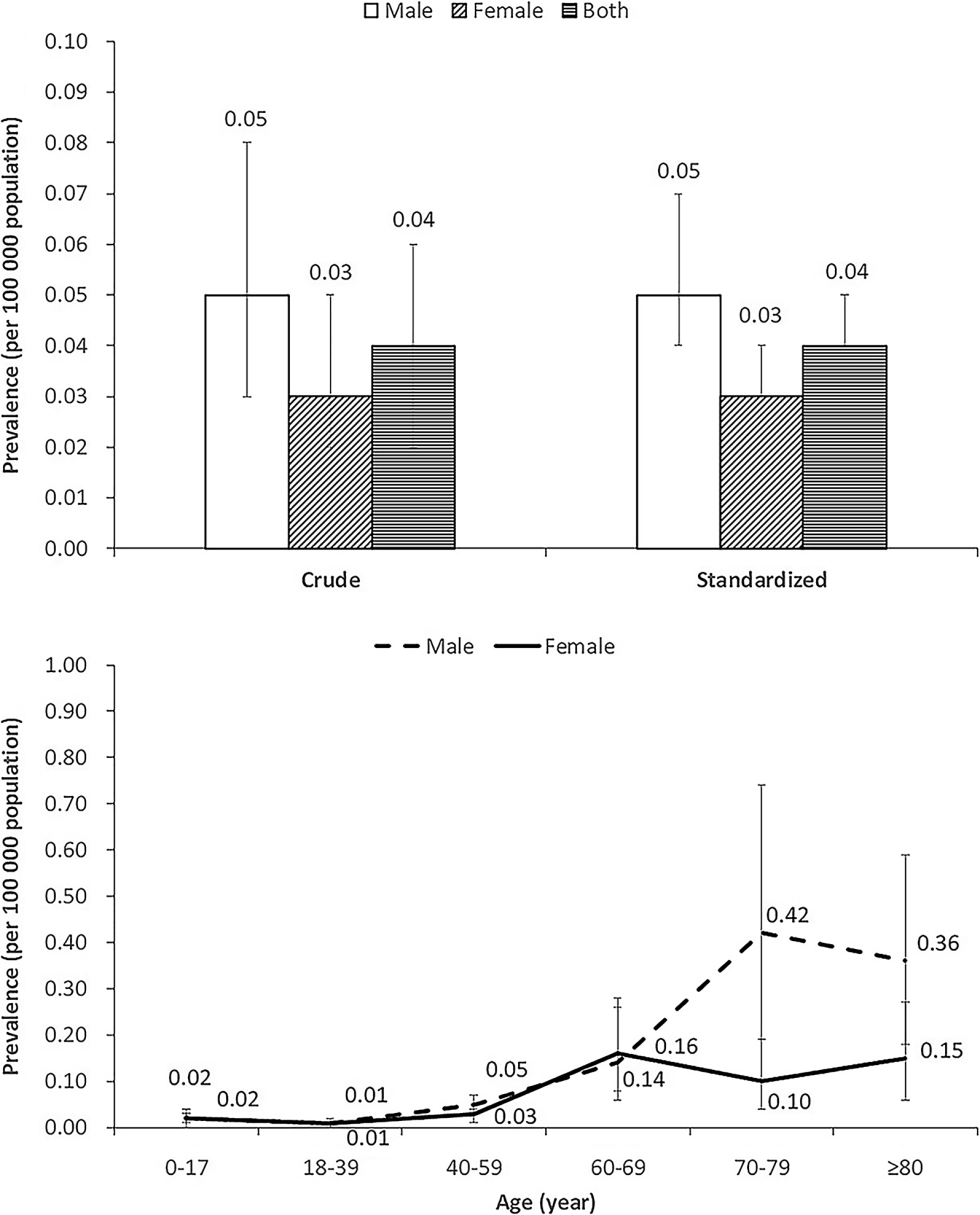
Prevalence of EMPD in urban China in 2016. *EMPD* extramammary Paget's disease. Note: The standardized prevalences are based on 2010 Chinese census data

The 2016 prevalence of EMPD in urban China in 2016 was 0.04 per 100,000 population (95% CI 0.02–0.06). Male prevalence (0.05, 95% CI 0.03–0.08) was higher than the female prevalence (0.03, 95% CI 0.02–0.05) (Fig. 1). In terms of the age trend of EMPD prevalence, the prevalence reached a peak at 70–79 years old (0.28, 95% CI 0.16–0.42) (Fig. 1). The EMPD prevalence in different regions varied, with the rates in Northwest China (0.07, 95% CI 0.02–0.16), Southcentral China (0.06, 95% CI 0.02–0.11) and Southwest China (0.08, 95% CI 0.03–0.16) were higher than other regions (Tables 3, 4).

**Table 3:** Unadjusted prevalence of EMPD in urban China in 2016 (unit: /100,000 population)

	Prevalence (95% CI)
Total	0.04 (0.02–0.06)
Sex	
Male	0.05 (0.03–0.08)
Female	0.03 (0.02–0.05)
Age group	
0–17	0.02 (0.01–0.03)
18–39	0.01 (0.00–0.01)
40–59	0.04 (0.02–0.05)
60–69	0.15 (0.09–0.23)
70–79	0.28 (0.16–0.42)
≥ 80	0.22 (0.13–0.33)
Area	
East	0.04 (0.02–0.08)
North	0.01 (0.00–0.02)
Northeast	0.01 (0.00–0.02)
Northwest	0.07 (0.02–0.16)
Southcentral	0.06 (0.02–0.11)
Southwest	0.08 (0.03–0.16)

*EMPD* extramammary Paget's disease, *CI* confidence interval

**Table 4:** Unadjusted prevalence of EMPD in urban China in 2016, by sex (unit: /100,000 population)

	Male (95% CI)	Female (95% CI)
Age group		
0–17	0.02 (0.00–0.03)	0.02 (0.01–0.04)
18–39	0.01 (0.00–0.01)	0.01 (0.00–0.02)
40–59	0.05 (0.03–0.07)	0.03 (0.01–0.04)
60–69	0.14 (0.06–0.26)	0.16 (0.08–0.28)
70–79	0.42 (0.19–0.74)	0.10 (0.04–0.19)
≥ 80	0.36 (0.18–0.59)	0.15 (0.06–0.27)
Area		
East	0.05 (0.02–0.09)	0.04 (0.01–0.10)
North	0.01 (0.00–0.03)	0.01 (0.00–0.04)
Northeast	0.00 (0.00–0.02)	0.01 (0.00–0.03)
Northwest	0.09 (0.02–0.20)	0.07 (0.00–0.22)
Southcentral	0.09 (0.01–0.22)	0.03 (0.01–0.05)
Southwest	0.08 (0.02–0.18)	0.08 (0.00–0.28)

*EMPD* extramammary Paget's disease, *CI* confidence interval

### Adjusted prevalence

The age-adjusted national prevalence based on 2010 Chinese census data was 0.04 per 100,000 population (95% CI 0.04–0.05). The standardized prevalence rates by the US, European and Australian populations were 0.05 (95% CI 0.05–0.06), 0.07 (95% CI 0.06–0.08) and 0.06 (95% CI 0.05–0.06), respectively.

### Sensitivity analysis

The lower bound of the national prevalence was calculated as 0.03 per 100,000 population (95% CI 0.02–0.04) by only considering the observed patients. The prevalence rates calculated by setting a stricter algorithm to identify patients with EMPD or excluding the top 10% of provinces with missing diagnostic information (i.e., Shandong and Xinjiang) were 0.04 (95% CI 0.02–0.06) and 0.05 (95% CI 0.03–0.07), respectively.

## Discussion

In this national study, we elucidated three primary findings. First, the crude prevalence of EMPD in mainland China was approximately 0.04 per 100,000 population in 2016. The age-adjusted prevalences of EMPD based on the 2010 Chinese national census data, the RESP 2013, the 2010 US population and the 2011 Australian population were all similar to the unadjusted prevalence in this study. This may be due to the fact that as a rare disease, even if the absolute figure of prevalence doubles, the variation in the prevalence was little and hard to be observed. In terms of China, the similar prevalence before and after adjustment based on the 2010 Chinese national census is due to the fact that the age structure of our study population is similar to that of the 2010 Chinese national census. So far, no other studies have ever reported the prevalence of EMPD. The incidence of EMPD ranged from 0.054 patients per 100,000 person-years to 0.11 patients per 100,000 person-years in Europe based on analysis of registry database [12, 13, 18]. In the United States, the incidence was 0.07 per 100,000 person-years in Caucasian males [1]. The prognosis of EMPD in those areas was relatively good, with reported five-year survival rates ranging from 50 to 98%. Since prevalence approximately equals the product of incidence and disease duration for cancers [20, 21], with the disease duration of EMPD ranging from 11 to 30 years [11], the prevalence in European countries or the United States should range from 0.6 per 100,000 population to 3.3 per 100,000 population, which was higher than the prevalence in China [11, 13, 22, 23]. Within Asian area, our result was approximated to the range of rates in Taiwan of China [24]. Therefore, we could not exclude the possibility that ethnic disparity might contribute to this discrepancy, considering the fact that skin cancers are more prevalent in whites than in Asians [25].

In our study, the prevalence of EMPD varied greatly by the geographic areas of mainland China. The Northwest China, Southcentral China and Southwest China presented significantly higher prevalence of EMPD than the rest of the areas. Based on current data, it is difficult for us to determine the exact explanation for this finding. However, this finding, to some extent, excluded the influences of socioeconomic level and medical level on the prevalence of EMPD, as the Northwest China and Southwest China are relatively underdeveloped areas in mainland China. In Europe, a clear difference in the incidence of EMPD among geographic areas were also reported, but the cause remained unclear [13]. In addition, no other studies accessing regional difference of EMPD were available to confirm our findings, further studies are needed to explain this regional discrepancy.

Secondly, patients with EMPD showed a male predominance in mainland China, with a male-to-female ratio of 1.7:1 in our study. This was consistent with multicenter studies in Asian population, the reported male-to-female ratios were approximately 3.5:1 in Taiwan of China [24], 3.9:1 in South Korea [26] and 2:1 in Japan [17]. By contrast, a significant female predominance was reported in studies from Western countries based on registry database, the male-to-female ratios ranged from 1:1.6 to 1:3.6 [9–13]. Consistent with these gender discrepancies, Asian studies commonly identify the scrotum and penis as the most frequent sites of involvement [26–29], rather than vulva, which is the most common site in Western populations [10, 13]. The reasons for this discrepancy remained unclear, there were two possible explanations. First, ethnic disparity might play a role, considering that Asian males were more susceptible to EMPD than Caucasian males in multiracial country [1]. Moreover, conservative attitudes among elderly Asian females that might discourage them from seeking medical treatment for lesions in the genital area was also considered an explanation in a previous study [24].

Thirdly, EMPD were more prevalent in aged patients older than 60, with a peak prevalence at 70–79 years of age in mainland China. This was consistent with previous literatures—that is, EMPD were commonly occurred in older individuals aged 60–80 years [8, 12, 14, 30]. The reason for the aging of EMPD patients was unclear, a more pro-oncogenic microenvironment in aged skin might be a possible explanation [31, 32]. In addition, we found that patients with EMPD in this study were much younger than those in the United States, Europe, Japan, South Korea and Taiwan of China [9–13, 17, 24, 26]. These areas listed above had a relatively longer life expectancy than that in mainland China, which suggested that the age for EMPD patients might be closely related to the mean life expectancy in corresponding areas [33]. However, the influence of ethnic differences should also be noted, as patients with EMPD in other Asian areas including Japan, South Korea, Thailand and Taiwan of China were consistently younger than those in Western populations [17, 24, 26, 30]. Further studies are needed to clarify this point in depth.

The large, national representative sample of Chinese urban population in this study not only ensured the overall estimation of the prevalence of a rare disease like EMPD but also allowed us to explore age and gender patterns of the prevalence as well as regional differences in China. This study has several limitations. First, the basic medical insurance database lacked some detailed information, such as tumor stage and laboratory results. It limited the possibility to stratify the diagnosis in greater detail. Second, rural inhabitants and certain urban populations, such as military soldiers are not included in the UEBMI and URBMI system because they have different types of medical insurance. The exclusion of these groups could have affected the estimates.

## Conclusions

This research is the first population-based study to investigate the prevalence of patients with EMPD in mainland China based on the basic medical insurance database. The prevalence of EMPD was lower in mainland China than those in Europe and the United States. Patients with EMPD in China were younger than those in developed countries, with a significant male predominance. These findings add to our understanding of the epidemiologic characteristics of EMPD in China. At the same time, as China is a country with a population of 1.3 billion, these findings can also provide important implications for further epidemiological studies of EMPD worldwide.

## Data Availability

The datasets generated and/or analysed during the current study are not publicly available due to privacy, but are available from the corresponding author on reasonable request.

## Abbreviations

EMPD: Extramammary Paget’s disease
UEBMI: Urban employee basic medical insurance
URBMI: Urban residence basic medical insurance
ICD: International classification of diseases
RESP: Revised European standard population
SD: Standard deviation
CI: Confidence interval

## References

[1] Herrel LA, Weiss AD, Goodman M (2015). Extramammary Paget's disease in males: survival outcomes in 495 patients. Ann Surg Oncol.

[2] Lam C, Funaro D (2010). Extramammary Paget's disease: Summary of current knowledge. Dermatol Clin.

[3] Asel M, LeBoeuf NR (2019). Extramammary Paget's disease. Hematol Oncol Clin North Am.

[4] Kanitakis J (2007). Mammary and extramammary Paget's disease. J Eur Acad Dermatol Venereol.

[5] Terlou A, Blok LJ, Helmerhorst TJM, van Beurden M (2010). Premalignant epithelial disorders of the vulva: squamous vulvar intraepithelial neoplasia, vulvar Paget's disease and melanoma in situ. Acta Obstet Gynecol Scand.

[6] Fan L, Zhu J, Tao X, Xu C (2016). Intraepithelial extramammary Paget's disease of the vulva: the clinicopathological characteristics, management, and outcome in a study of 18 female patients. Dermatol Surg.

[7] Morris CR, Hurst EA (2020). Extramammary Paget disease: a review of the literature-part i: history, epidemiology, pathogenesis, presentation, histopathology, and diagnostic work-up. Dermatol Surg.

[8] Simonds RM, Segal RJ, Sharma A (2019). Extramammary Paget's disease: a review of the literature. Int J Dermatol.

[9] Lee GC, Kunitake H, Stafford C, Bordeianou LG, Francone TD, Ricciardi R (2019). High risk of proximal and local neoplasms in 2206 patients with anogenital extramammary Paget's disease. Dis Colon Rectum.

[10] Yao H, Xie M, Fu S (2018). Survival analysis of patients with invasive extramammary Paget disease: implications of anatomic sites. BMC cancer.

[11] Karam A, Dorigo O (2012). Treatment outcomes in a large cohort of patients with invasive Extramammary Paget's disease. Gynecol Oncol.

[12] Siesling S, Elferink MAG, van Dijck JAAM, Pierie JPEN, Blokx WAM (2007). Epidemiology and treatment of extramammary Paget disease in the Netherlands. Eur J Surg Oncol.

[13] van der Zwan JM, Siesling S, Blokx WAM, Pierie JPEN, Capocaccia R (2012). Invasive extramammary Paget's disease and the risk for secondary tumours in Europe. Eur J Surg Oncol.

[14] Lee K-Y, Roh MR, Chung WG, Chung KY (2009). Comparison of Mohs micrographic surgery and wide excision for extramammary Paget's Disease: Korean experience. Dermatol Surg.

[15] Urabe A, Matsukuma A, Shimizu N, Nishimura M, Wada H, Hori Y (1990). Extramammary Paget's disease: comparative histopathologic studies of intraductal carcinoma of the breast and apocrine adenocarcinoma. J Cutan Pathol.

[16] Chang YT, Liu HN, Wong CK (1996). Extramammary Paget's disease: a report of 22 cases in Chinese males. J Dermatol.

[17] Hatta N, Yamada M, Hirano T, Fujimoto A, Morita R (2008). Extramammary Paget's disease: treatment, prognostic factors and outcome in 76 patients. Br J Dermatol.

[18] Ghazawi F, Le M, Alakel A, Rahme E, Litvinov IV (2019). The epidemiology and clinical characteristics of extramammary Paget disease patients in Canada and assessing the risk of second malignancies. J Invest Dermatol.

[19] Wang S, Xu L, Feng J (2019). Prevalence and incidence of multiple myeloma in urban area in China: a national population-based analysis. Front Oncol.

[20] Freeman J, Hutchison GB (1980). Prevalence, incidence and duration. Am J Epidemiol.

[21] Preston SH (1987). Relations among standard epidemiologic measures in a population. Am J Epidemiol.

[22] van der Linden M, Oonk MHM, van Doorn HC (2019). Vulvar Paget disease: a national retrospective cohort study. J Am Acad Dermatol.

[23] Nasioudis D, Bhadra M, Ko EM (2020). Extramammary Paget disease of the vulva: management and prognosis. Gynecol Oncol.

[24] Cheng PS, Lu CL, Cheng CL, Lai FJ (2014). Significant male predisposition in extramammary Paget disease: a nationwide population-based study in Taiwan. Br J Dermatol.

[25] Agbai ON, Buster K, Sanchez M (2014). Skin cancer and photoprotection in people of color: a review and recommendations for physicians and the public. J Am Acad Dermatol.

[26] Lee S-J, Choe YS, Jung HD (2011). A multicenter study on extramammary Paget's disease in Korea. Int J Dermatol.

[27] Kang Z, Zhang Q, Zhang Q (2015). Clinical and pathological characteristics of extramammary Paget's disease: report of 246 Chinese male patients. Int J Clin Exp Pathol.

[28] Shiomi T, Yoshida Y, Yamamoto O, Umekita Y (2015). Extramammary Paget's disease: evaluation of the adnexal status of 53 cases. Pol J Pathol.

[29] Qi Y, Hu J, Sun C, Zhang J, Liu Q (2014). Extramammary Paget's disease: analysis of 17 Chinese cases. Indian J Dermatol Venereol Leprol.

[30] Ito Y, Igawa S, Ohishi Y, Uehara J, Yamamoto AI, Iizuka H (2012). Prognostic indicators in 35 patients with extramammary Paget's disease. Dermatol Surg.

[31] Smetana K, Lacina L, Szabo P, Dvořánková B, Brož P, Šedo A (2016). Ageing as an important risk factor for cancer. Anticancer Res.

[32] Hayes C, Defeo K, Shieh C, Trempus C, Dang H, Gilmour S (2008). Age-related stromal alterations in the skin that facilitate tumor development. Cancer Res.

[33] GBD 2016 Mortality Collaborators (2017). Global, regional, and national under-5 mortality, adult mortality, age-specific mortality, and life expectancy, 1970–2016: a systematic analysis for the Global Burden of Disease Study 2016. Lancet.

